# Borna Disease Virus Blocks Potentiation of Presynaptic Activity through Inhibition of Protein Kinase C Signaling

**DOI:** 10.1371/journal.ppat.0020019

**Published:** 2006-03-17

**Authors:** Romain Volmer, Céline Monnet, Daniel Gonzalez-Dunia

**Affiliations:** 1 Avenir group, INSERM, U563, Toulouse, France; 2 Unité des Virus Lents, CNRS URA 1930, Département de Virologie, Institut Pasteur, Paris, France; Scripps Research Institute, United States of America

## Abstract

Infection by Borna disease virus (BDV) enables the study of the molecular mechanisms whereby a virus can persist in the central nervous system and lead to altered brain function in the absence of overt cytolysis and inflammation. This neurotropic virus infects a wide variety of vertebrates and causes behavioral diseases. The basis of BDV-induced behavioral impairment remains largely unknown. Here, we investigated whether BDV infection of neurons affected synaptic activity, by studying the rate of synaptic vesicle (SV) recycling, a good indicator of synaptic activity. Vesicular cycling was visualized in cultured hippocampal neurons synapses, using an assay based on the uptake of an antibody directed against the luminal domain of synaptotagmin I. BDV infection did not affect elementary presynaptic functioning, such as spontaneous or depolarization-induced vesicular cycling. In contrast, infection of neurons with BDV specifically blocked the enhancement of SV recycling that is observed in response to stimuli-induced synaptic potentiation, suggesting defects in long-term potentiation. Studies of signaling pathways involved in synaptic potentiation revealed that this blockade was due to a reduction of the phosphorylation by protein kinase C (PKC) of proteins that regulate SV recycling, such as myristoylated alanine-rich C kinase substrate (MARCKS) and Munc18–1/nSec1. Moreover, BDV interference with PKC-dependent phosphorylation was identified downstream of PKC activation. We also provide evidence suggesting that the BDV phosphoprotein interferes with PKC-dependent phosphorylation. Altogether, our results reveal a new mechanism by which a virus can cause synaptic dysfunction and contribute to neurobehavioral disorders.

## Synopsis

The central nervous system is the target of many persistent viral infections that can induce diverse pathological manifestations. Besides causing meningitis or encephalitis, viruses can infect neurons without overt structural damage, but nevertheless alter cellular functioning by yet-undefined molecular mechanisms, thereby disturbing homeostasis and causing disease. Here, the authors have studied the infection by Borna disease virus, an RNA virus that persists in the brain of a wide variety of animals and causes behavioral disturbances. Using primary cultures of neurons, they show that Borna disease virus interferes specifically with the activity-dependent enhancement of synaptic activity, one form of synaptic plasticity that is believed to be essential for memory formation. This interference was correlated to a reduced phosphorylation of neuronal targets by protein kinase C (PKC), a kinase that plays important roles in the regulation of neuronal activity. The authors also provide evidence that the viral phosphoprotein may be responsible for this interference, possibly by competing with the phosphorylation of endogenous cellular PKC substrates. These results illustrate an intriguing aspect of viral interference with neuronal function and reveal a new mechanism whereby a virus can cause synaptic dysfunction and contribute to neurobehavioral disorders.

## Introduction

Viruses can affect brain functioning in several ways. In some cases, viral replication causes neuronal death directly, as in the manner of rabies virus or alphaviruses, which induce neuronal apoptosis [1,2]. Alternatively, neurons can be damaged by immune cytotoxicity or by neurotoxic factors produced by infiltrating mononuclear cells or infected glial cells [3]. Viruses can also persist in neurons and cause neurological diseases without overt cytopathic effect or inflammation [4]. This has led to the hypothesis that persistent viruses could play a role in human mental disorders of unclear etiology [5,6]. It also has provided further impetus to understand the molecular mechanisms underlying virus-induced neuronal dysfunction.

Borna disease virus (BDV) is an attractive paradigm for investigating the mechanisms of neurobehavioral disorders due to the persistence of a non-cytolytic virus. BDV is an enveloped virus with a non-segmented, negative strand RNA genome [7,8]. BDV infects a wide variety of mammals [9], and serological evidence suggests that BDV, or a BDV-like virus, also infects humans [10,11]. Infected hosts develop a large spectrum of neurological disorders, ranging from immune-mediated diseases to behavioral alterations without inflammation [9,12], reminiscent of symptoms observed in human psychiatric diseases such as schizophrenia, mood disorders, and autism [13]. These neurobehavioral manifestations reflect the remarkable localization of BDV in the central nervous system (CNS). The virus targets mainly neurons of the limbic system and persists primarily in the hippocampus [14].

The molecular bases for the cognitive impairment of BDV-infected animals remain to be determined. Since BDV is non-cytolytic, it was suggested that BDV interferes with signaling pathways that are important for proper neuronal functioning [5,15]. This hypothesis was corroborated by the observation that BDV reduces the expression of proteins involved in synaptic remodeling [16] and blocks the response of neurons to neurotrophins [17]. However, no study has tested directly the impact of BDV infection on synaptic transmission and on activity-dependent plasticity.

Synaptic transmission is initiated by the release of neurotransmitters through exocytosis of synaptic vesicles (SV). Once they have fused at the plasma membrane, SV are endocytosed and refilled with neurotransmitters. This cycle of exo-endocytosis, termed SV recycling, allows SV to be reused for subsequent neurotransmitter release. SV recycling is essential to maintain efficient synaptic transmission and is tightly regulated [18]. In recent years, evidence has accumulated showing that changes in the rate of SV recycling modulate synaptic activity and contribute to synaptic plasticity. For example, it has been shown that vesicular cycling increases markedly during long-term potentiation (LTP), a form of synaptic plasticity that is thought to underlie forms of learning and memory at the cellular level [19,20]. It is also conceivable that a loss of proper regulation of SV exo-endocytosis could contribute to cognitive and behavioral impairment associated with BDV infection.

The goal of the present work was to examine the impact of BDV infection on synaptic activity, by analyzing SV recycling. We used infected primary cultures of hippocampal neurons and measured the rate of vesicular cycling under spontaneous conditions or after different stimulation procedures. Here, we report that BDV selectively blocks activity-dependent enhancement of SV recycling, whereas it does not affect spontaneous or depolarization-induced vesicular cycling. We also analyzed signaling pathways that play important roles for presynaptic potentiation and found that this blockade is linked to an interference with protein kinase C (PKC) signaling. Finally, we show that transfection of BDV phosphoprotein (P) in neurons leads to impaired PKC-dependent phosphorylation. Thus, we provide a cellular and molecular basis for the learning disabilities associated with BDV infection.

## Results

### Synapse Integrity and Basal and Evoked Presynaptic Activities Are Unaffected by BDV

**Figure 1:**
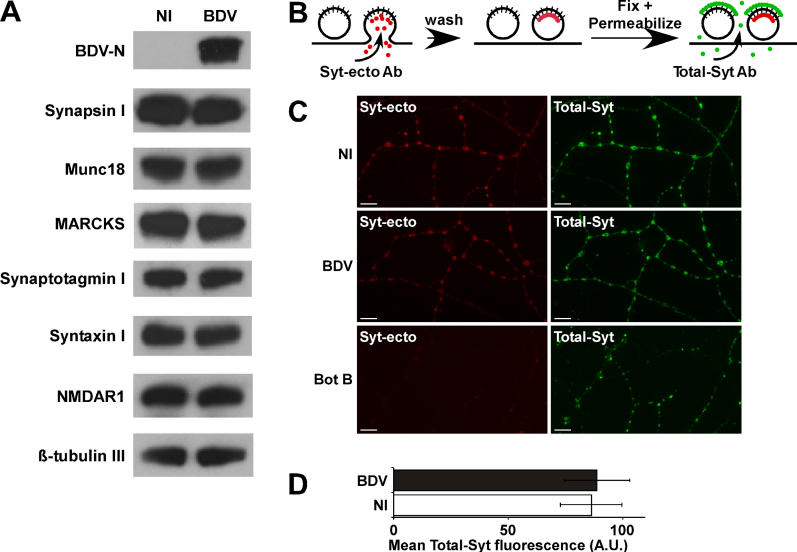
Synapse Integrity Is Preserved in Neurons Persistently Infected with BDV (A) Western blots of neuronal extracts. Comparative analysis of the expression levels of a panel of neuronal proteins from non-infected (NI) and BDV-infected cultures. Results are representative of three independent experiments. (B) Outline of the experimental protocol to measure vesicular cycling at synapses. In a first step, recycling vesicles are labeled with a mouse monoclonal antibody directed against the luminal domain of synaptotagmin 1 (Syt-ecto). After washes, neurons are fixed and permeabilized and incubated with a rabbit antibody directed against the cytoplasmic domain of synaptotagmin 1 (Total-Syt). (C) Examples of Syt-ecto and total-Syt labeling. Bottom panels show neurons treated with botulinum B toxin (Bot B). Bar: 20 μm. (D) Quantification of total-Syt fluorescence staining (*n* = 381 and 386 synapses for BDV-infected and NI neurons, respectively).

We used hippocampal neurons which had been cultured and infected for 14 d. At that time point, the neurons have established mature synapses and the virus has spread to all of them [21,22]. No differences were found in the expression of proteins involved in synaptic transmission (Figure 1A), confirming that BDV infection does not impair synaptic protein expression [17]. To study whether BDV replication affected synaptic activity, we analyzed SV recycling with an assay based on the uptake of a monoclonal antibody targeted against the luminal domain of the vesicular protein synaptotagmin I (Syt-ecto; Figure 1B and 1C). Living neurons were first incubated with Syt-ecto in order to specifically label vesicles that have fused at the plasma membrane. Thereafter, the cultures were fixed and stained with a polyclonal antibody recognizing the cytoplasmic domain of synaptotagmin I (total-Syt). After image acquisition, the regions of interest corresponding to synapses were identified in the total-Syt channel (detected by an anti-rabbit FITC, fluorescence F2). Fluorescence intensity in the Syt-ecto channel (using an anti-mouse-Cy3, fluorescence F1) was quantified within the same region. We then calculated the ratio of fluorescence intensities F1/F2 to obtain a normalized measurement of SV recycling. The specificity of Syt-ecto (F1) staining was demonstrated by loss of labeling after overnight treatment with botulinum B toxin, which cleaves VAMP-2 protein and abrogates SV recycling [23] (Figure 1C, bottom panels). To avoid any bias in the measurements of fluorescence ratios, we also verified that total-Syt fluorescence (F2) did not differ between control and BDV-infected neurons (Figure 1D), consistent with Western blot results for synaptotagmin I shown on Figure 1A.

**Figure 2:**
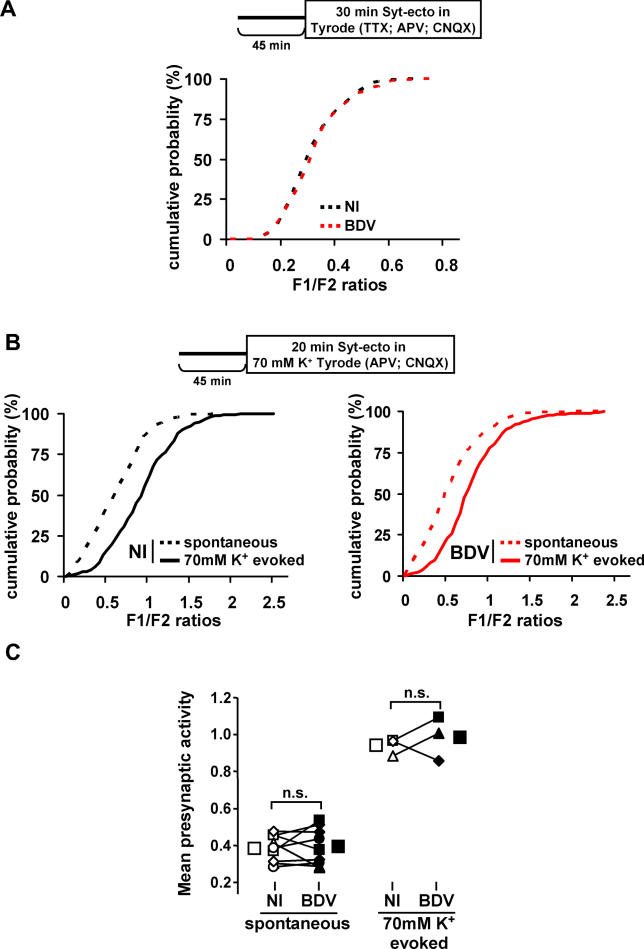
Spontaneous and Evoked SV Recycling Are Not Affected by BDV Infection (A) Cumulative probability distributions of fluorescence ratios (F1/F2) for individual synapses for spontaneous SV recycling. No difference is observed between control (NI) and BDV-infected neurons (*p* = 0.112, KS test). (B) Cumulative probability distributions of fluorescence ratios (F1/F2) for individual synapses for 70 mM K^+^ evoked and spontaneous SV recycling. In both NI and BDV-infected neurons, the level of SV recycling is significantly increased in neurons stimulated with 70 mM K^+^ (*p* < 0.001, KS test). The overview of the experimental stimulation protocol is depicted above each graph. (C) Pairwise comparisons of mean presynaptic activities in independent experiments involving at least 300 synapses (small symbols). Bigger squares correspond to mean values of all independent experiments. n.s., not significant using paired *t* test.

In a first set of experiments, we measured basal presynaptic activity in the presence of blockers of endogenous neuronal activity, i.e., TTX, APV, and CNQX, so that SV recycling is due to spontaneous fusion and recovery of vesicles, without firing of action potentials or activity due to endogenous glutamate release (Figure 2) [20]. Under these conditions, the level of SV recycling was low and did not differ between control and BDV-infected neurons (Figure 2A and 2C). Likewise, when neurons were subjected to an extended depolarization, we observed the same enhancement of SV recycling for control and BDV-infected neurons, likely due to the mobilization of the entire pool of recycling SV (Figure 2B and 2C) [24]. Together, these data indicate that infection with BDV has no impact on basal presynaptic activity or on the ability to respond to a prolonged depolarization.

### BDV Impairs Activity-Dependent Potentiation of Vesicular Recycling

**Figure 3:**
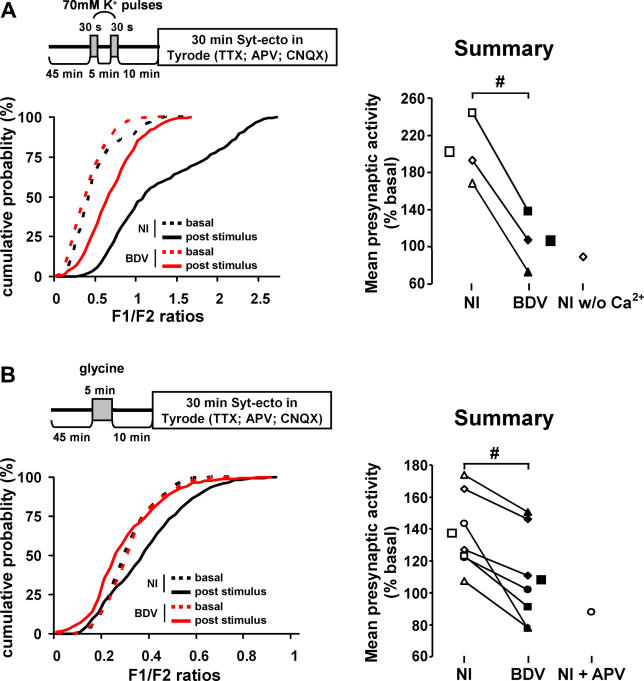
BDV Blocks Activity-Dependent Potentiation of Vesicular Recycling Cumulative probability distributions of fluorescence ratios (F1/F2) for individual synapses for the analysis of SV recycling in spontaneous conditions and after: (A) repetitive depolarization with high-K^+^ pulses or (B) glycine treatment. The overview of the experimental stimulation protocol is depicted above each graph. The level of SV recycling is significantly lower in BDV-infected neurons compared to NI neurons following stimulation with high-K^+^ pulses or glycine treatment (*p* < 0.001 using KS test). Note that SV recycling is even reduced after glycine treatment in BDV-infected neurons. Pairwise comparisons of mean presynaptic activities in independent experiments involved at least 300 synapses (small symbols). Bigger squares correspond to mean values of all independent experiments. Similar results were found in three to seven independent experiments. Hash mark (#) indicates *p* < 0.05 by paired *t* test.

Previous studies have demonstrated that an increase of SV recycling is important for certain forms of synaptic plasticity, and in particular for the expression of LTP [19,20]. These findings prompted us to ask whether BDV replication impacted the enhancement of SV recycling observed in response to stimuli-induced synaptic potentiation. Indeed, after repetitive depolarization using high K^+^ pulses [25], we observed that SV recycling was strongly enhanced in control neurons, but only modestly in BDV-infected neurons (Figure 3A). No enhancement of SV recycling was observed when neurons were stimulated in the absence of calcium, confirming that calcium influx is necessary to trigger potassium-induced potentiation. Similar results were found when neurons were stimulated with glycine, which has been shown to induce LTP in hippocampal neurons in culture [26,27]. An increase in SV recycling was detected after washout of glycine in control neurons, whereas no enhancement (and even a statistically significant reduction) was observed in BDV-infected neurons (Figure 3B). The increase in SV recycling in control neurons was prevented when the N-methyl-D-aspartate (NMDA) receptor blocker APV was added, consistent with the requirement of NMDA receptor activation for glycine-induced LTP [26]. Note that glycine, a milder stimulus than high K^+^ pulses, led to less pronounced enhancement of SV recycling in control neurons. Thus, BDV infection interferes with activity-dependent changes in presynaptic activity induced by two different stimulation procedures.

### BDV Interferes with PKC-Dependent Synaptic Potentiation

**Figure 4:**
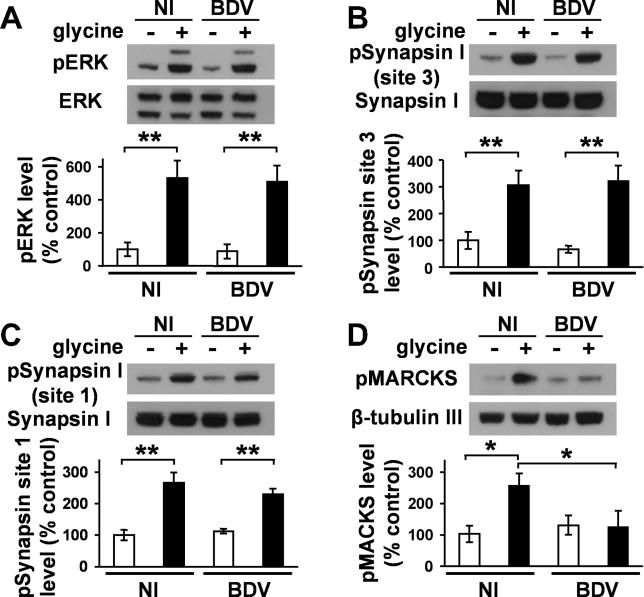
PKC Signaling Is Impaired in BDV-Infected Neurons Western blots of neuronal extracts from NI and BDV-infected cultures stimulated with glycine using antibodies specific for: (A) Diphosphorylated ERK 1/2 (pERK) and total ERK 1/2; (B) phospho-synapsin I (pSynapsin I; Site 3, a site specific for CaMK II), and total synapsin I; (C) phospho-synapsin I (Site 1, a site specific for PKA and CamK I) and total synapsin I; (D) phospho-MARCKS (pMARCKS; PKC site at Ser152/156) and β-tubulin III. Results are representative of four independent experiments. Single asterisk (*) indicates *p* < 0.05; double asterisks (**) indicate *p* < 0.01 by unpaired *t* test.

Signaling by several protein kinases plays important roles during the early phase of synaptic potentiation. In particular, extracellular-regulated kinase (ERK) 1/2, protein kinase A (PKA), Ca^2+^/calmodulin-dependent kinase (CaMK) II, and PKC phosphorylate presynaptic proteins that modulate SV recycling [28,29]. Thus, we tested the hypothesis that BDV-mediated block in the enhancement of SV recycling could be due to an interference with protein kinase signaling, by using Western blot analyses with phospho-specific antibodies. Following stimulation of neurons with glycine, we observed that ERK 1/2 phosphorylation was not affected by BDV infection (Figure 4A). In addition, staining for synapsin I phosphorylated at either site 3 or site 1, two sites specific for CaMK II and PKA/CaMK I, respectively [30], showed that BDV did not interfere with signaling by these kinases (Figure 4B and 4C). In contrast, the phosphorylation of myristoylated alanine-rich C kinase substrate (MARCKS), a major PKC substrate in neurons [31], was severely impaired in BDV-infected neurons following treatment with glycine (Figure 4D). The phosphorylation of MARCKS was also impaired in BDV-infected neurons following exposure to high K^+^ pulses, although we observed a higher variability between experiments, likely due to the stronger effects of this stimulation procedure compared to glycine (unpublished data).

**Figure 5:**
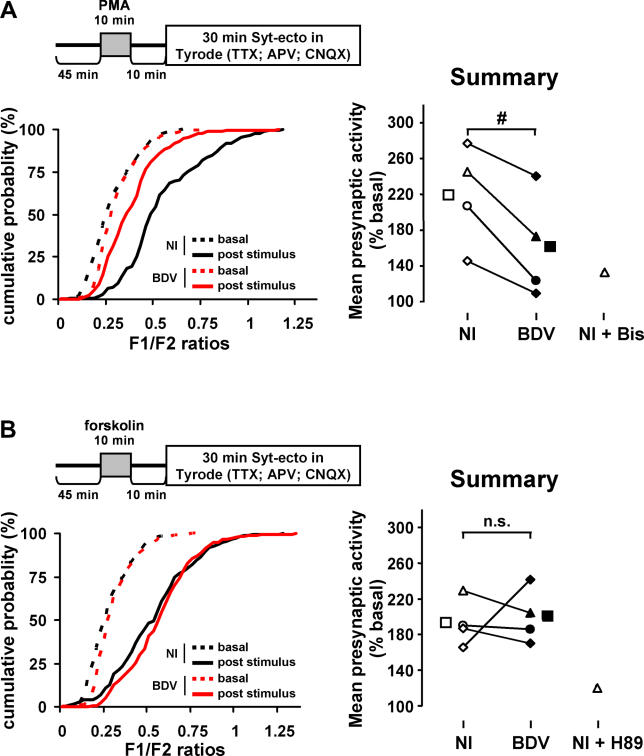
PMA, but Not Forskolin-Induced Potentiation of Vesicular Recycling Is Impaired in BDV-Infected Neurons Cumulative probability distributions of fluorescence ratios (F1/F2) for individual synapses for the analysis of SV recycling in spontaneous conditions and after (A) PMA treatment or (B) forskolin treatment. The overview of the experimental stimulation protocol is depicted above each graph. The level of SV recycling is significantly lower in BDV-infected neurons compared to NI neurons after stimulation with PMA (*p* < 0.001 using KS test). Pairwise comparisons of mean presynaptic activities in independent experiments involved at least 180 synapses (small symbols). Bigger squares correspond to mean values of all independent experiments. Similar results were found in four independent experiments. Also shown are results for NI neurons treated with the PKC inhibitor Bis or the PKA inhibitor H89. Hash mark (#) indicates *p* < 0.05. n.s., not significant, by paired *t* test.

To further determine the level of BDV interference with PKC signaling, we investigated whether BDV interference with synaptic potentiation was still observed after direct activation of PKC by phorbol 12-myristate 13-acetate (PMA). Phorbol esters, such as PMA, have been shown to enhance vesicular cycling through the presynaptic PKC/munc-13 signaling cascades [32,33]. Although treatment with PMA led to an increase in SV recycling in both control and BDV-infected neurons, the level of potentiation was much lower in BDV-infected neurons (Figure 5A). In neurons treated with the PKC inhibitor Bis, potentiation of presynaptic activity was almost completely abolished, indicating that the majority of the PMA-induced enhancement of vesicular cycling could indeed be attributed to PKC signaling. We also analyzed PKA-dependent enhancement of SV recycling by stimulating PKA directly with forskolin [34]. Consistent with the analysis of synapsin I phosphorylation at site 1 (Figure 4C), we observed that forskolin enhanced SV recycling to the same extent in control and BDV-infected neurons (Figure 5B).

**Figure 6:**
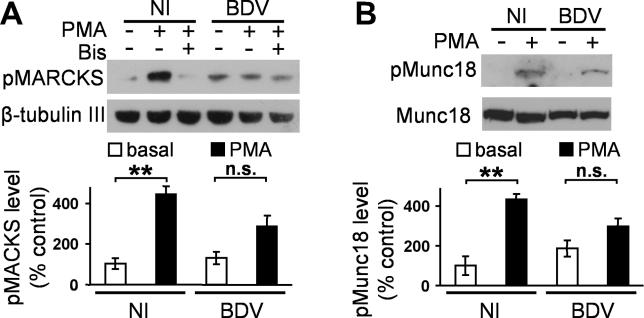
Analysis of PKC Signaling in NI and BDV-Infected Neurons Western blot analyses of (A) MARCKS and (B) Munc18 phosphorylation by PKC. β-Tubulin III and total Munc18 were used to normalize expression. Results are representative of three to five independent experiments. Double asterisks (**) indicate *p* < 0.01. n.s., not significant, using unpaired *t* test.

We then analyzed the phosphorylation of PKC substrates following direct stimulation of PKC by PMA. We showed by Western blot analysis that the phosphorylation of MARCKS and Munc18–1/nSec1 (Munc18), two PKC substrates that have been implicated in the regulation of vesicular recycling [31,35], was impaired in BDV-infected neurons following direct stimulation of PKC by PMA (Figure 6A and 6B).

### BDV Interferes with PKC Signaling Downstream of PKC Activation

**Figure 7:**
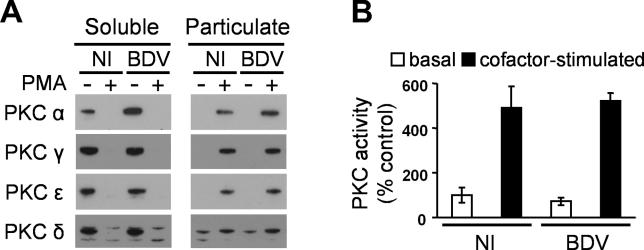
Analysis of PKC Activation (A) Cytosol-to-membrane recruitment of PKC. Subcellular distribution of PKC isoforms in NI and BDV-infected neurons after treatment with PMA. The detergent-soluble and particulate fractions, corresponding to cytosolic and membrane fractions, were subjected to Western blots for the detection of PKCα, PKCγ, PKCɛ, and PKCδ. Note the shift in the subcellular distribution of PKC isoforms after treatment with PMA. Results are representative of three independent experiments. (B) In vitro measurements of basal and cofactor-stimulated PKC activities. The cofactor-stimulated specific activity represents kinase activity in the presence of Ca^2+^ and lipid activators. Results are expressed as percentage of increase over the mean of unstimulated controls.

BDV could interfere with PKC signaling by inhibiting PKC recruitment to membranes, an essential step in PKC activation [36]. We therefore examined the cytosol-to-membrane translocation of PKC isoforms α, γ, ɛ, and δ [37] and showed that BDV infection did not interfere with membrane translocation of these PKC isoforms following PMA treatment (Figure 7A). In addition, both basal and cofactor-stimulated PKC activities, measured using an in vitro kinase assay, did not differ between control and BDV-infected neurons (Figure 7B). Thus, BDV infection does not alter the ability of PKC to be activated. Altogether, these data indicate that BDV interferes with PKC signaling downstream of PKC activation.

**Figure 8:**
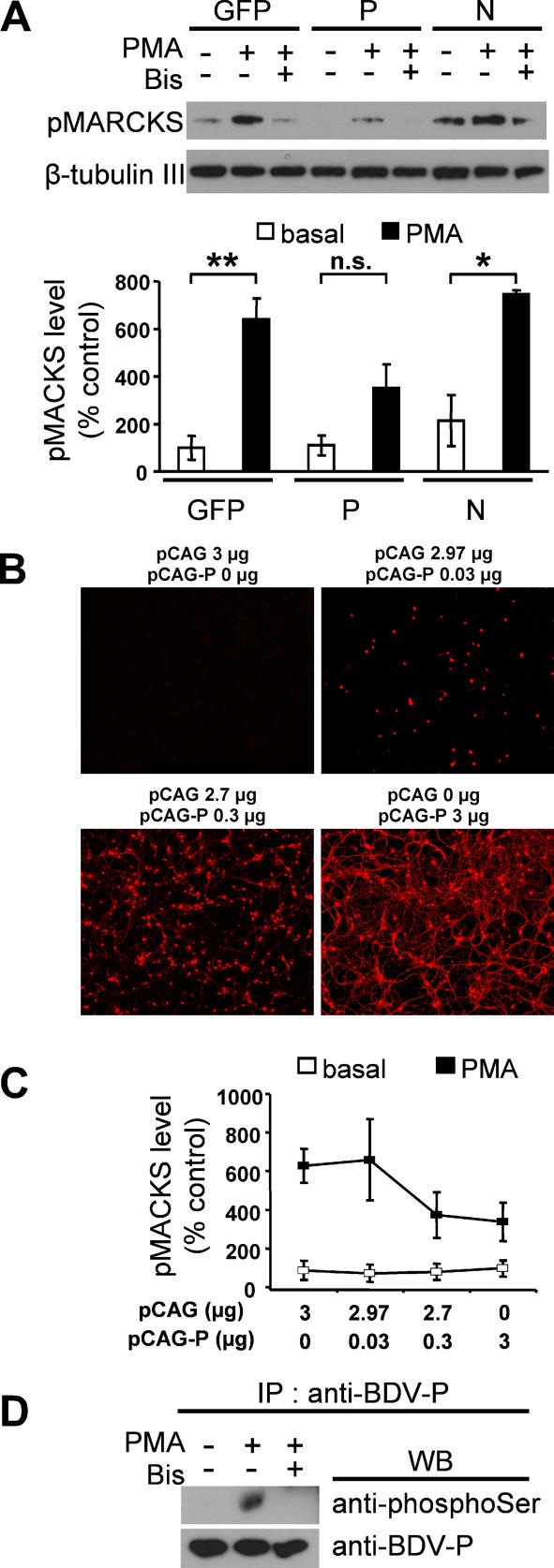
BDV P Interferes with PKC-Dependent Phosphorylation (A) Phosphorylation of MARCKS following treatment with PMA was analyzed in neurons transfected with expression vectors for GFP, BDV P, and BDV N. β-Tubulin III is used to normalize expression. Results are expressed as percentage of non-stimulated GFP-transfected neurons. (B) Immunofluorescence analysis of BDV P expression in neurons transfected with increasing quantities of the BDV P plasmid (pCAG-P). When necessary, the amount of pCAG-P plasmid was supplemented with empty plasmid (i.e., the same pCAG plasmid backbone without insertion of P), so that the total amount is 3 μg in all cases Note that BDV P is detected in neuronal processes, starting at 0.3 μg pCAG-P used in the transfection. (C) Dose-dependent interference of BDV P with PKC signaling. Phosphorylation of MARCKS following treatment with PMA was analyzed in neurons transfected with increasing quantities of BDV P plasmid (pCAG-P). The results are representative of four to seven independent experiments. Results are expressed as percentage of unstimulated neurons transfected with the empty vector. (D) BDV P is phosphorylated by PKC following treatment with PMA. BDV P was immunoprecipitated from BDV-infected neurons and subjected to Western blot analysis with a phospho-serine antibody. The membrane was then stripped and subjected to staining for BDV P. The phospho-serine positive band co-localizes with the band corresponding to P and is no longer detected in neurons preincubated with PKC inhibitor Bis. Single asterisk (*) indicates *p* < 0.05; double asterisks (**) indicate *p* < 0.01 by unpaired *t* test.

Previously, it has been shown that the BDV P is phosphorylated by PKC in vivo and in vitro [38]. In addition, P is strongly expressed in infected neurons. Thus, we hypothesized that P could compete with the phosphorylation of endogenous PKC substrates. In order to test this hypothesis, we transfected primary neurons with vectors expressing P, but also BDV nucleoprotein (N), the other strongly expressed viral protein, as well as green fluorescent protein (GFP) as a control. Expression of P was sufficient to decrease significantly the phosphorylation of MARCKS after stimulation with PMA, whereas N or GFP had no effect (Figure 8A). Transfection experiments using increasing quantities of P plasmid showed that this inhibitory effect on the phosphorylation of MARCKS was dose dependent (Figure 8B and 8C). Interestingly, the subcellular localization of BDV P varied depending on the amount of P plasmid used. Starting at 0.3 μg BDV P plasmid, we observed that P was detected not only in the nucleus, but also in neuronal processes (Figure 8B). We also showed that P was phosphorylated at serine residues following treatment with PMA (Figure 8D), in agreement with the previous demonstration that P is phosphorylated by PKC.

## Discussion

Here, we show that hippocampal neurons infected with BDV become unresponsive to synaptic potentiation. We further demonstrate that this phenotype is linked to an impairment of PKC signaling, and we provide evidence that BDV P interferes with the phosphorylation of endogenous PKC substrates in neurons. To our knowledge, this is the first demonstration of the direct effect of a viral infection on activity-dependent synaptic plasticity, representing an intriguing aspect of viral interference with neuronal functioning.

Remarkably, basal and evoked presynaptic activities were not affected in BDV-infected neurons. This correlates well with the total lack of cytopathic effect of BDV, which can infect primary neurons with high efficacy without impairing neuronal growth or expression of neuron-specific markers [17]. It also suggests that the active replication of BDV in neurons and the accumulation of viral proteins at synapses [22] does not lead to a generalized defect in presynaptic function.

The BDV-induced blockade in PKC signaling was also observed after direct activation of PKC by the agonist PMA. On the contrary, the PKA agonist forskolin induced similar enhancement of vesicular cycling in control and BDV-infected neurons, demonstrating that the cAMP/PKA signaling cascade is not targeted by infection. These findings underline the specificity of BDV interference with PKC-dependent potentiation and show that BDV-infected neurons are still capable of presynaptic potentiation, as long as PKC signaling is not required.

Viral interference with PKC signaling has often been correlated with the inhibition of the cytosol-to-membrane translocation of a specific PKC isozyme, due to the interaction of a viral protein with the anchoring protein RACK1 (receptor for activated PKC). Some examples of such an inhibition include the Nef protein from human immunodeficiency virus or the Zebra protein from Epstein-Barr virus [39,40]. In contrast, we showed here that the activation and membrane translocation of PKC isoforms were not affected by BDV infection. Rather, BDV blocked the phosphorylation of PKC neuronal substrates, such as MARCKS and Munc18, which play essential roles in vesicular cycling. The phosphorylation of MARCKS by PKC is implicated in actin-dependent cytoskeletal plasticity [41] and has been associated with PKC-mediated enhancement of neurotransmitter release, as well as with persistence of LTP in vivo [42]. Furthermore, PKC-dependent phosphorylation of the presynaptic protein Munc18 regulates neurotransmitter release and has been implicated in presynaptic plasticity [35].

Interestingly, inhibition of the phosphorylation of endogenous PKC substrates was also observed after transfection of neurons with BDV P. P, a cofactor for the viral polymerase complex, is abundantly expressed in the CNS following infection and was previously shown to be phosphorylated by PKC [38], consistent with our findings that P is phosphorylated at serine residues following PMA treatment. How could BDV P interfere with PKC-dependent phosphorylation in neurons? It is conceivable that the activity of PKC depends on the accessibility and the concentration of its substrates in the cell, and we postulate that the accumulation of a viral protein that can serve as a substrate for PKC may compete with the phosphorylation of endogenous PKC targets. Very recently, it was shown that BDV P can inhibit the Traf family member–associated NF-κB (TANK)-binding kinase-1 (TBK-1) in non–neuronal cells and that it may act as a decoy substrate, impairing the phosphorylation of endogenous TBK-1 substrates [43]. It would be interesting to test whether the same mechanism also applies to PKC-dependent signaling in neurons.

Expression of P in glial cells of transgenic mice leads to behavioral abnormalities [44]. Our results suggest that the expression of P in neurons, the main target cells for BDV persistence, is likely to cause neurobiological disturbances, through interference with PKC signaling. BDV replication is tightly regulated by complex mechanisms and the phosphorylation of P by PKC could increase the activity of the viral polymerase complex, thereby contributing to BDV adaptation to persistence in neurons [45,46]. We propose that the stimulation of synaptic connections of infected neurons, for example during learning, leads to increased phosphorylation of P consecutive to PKC activation and hence to increased viral replication and spread in the CNS, at the expense of behavioral disabilities for the host. This could also be one of the reasons for the preferential tropism of BDV for the hippocampus, a brain area subjected to important synaptic remodeling and characterized by strong PKC activity [38].

We previously described that BDV blocks ERK 1/2 activation in response to brain-derived neurotrophic factor (BDNF) [17]. This interference was demonstrated downstream of its tyrosine kinase receptor TrkB, but the precise link with impaired ERK activation remained to be identified. Here, we showed that the stimulation with glycine did not interfere with ERK 1/2 activation, suggesting that some pathways leading to ERK 1/2 activation are preserved following infection with BDV. It is well known that several intracellular pathways can mediate ERK phosphorylation in response to neurotrophins or other extracellular signals [47,48]. In addition to the linear Ras/cRaf/MEK/ERK pathway, members of the PKC family have also been shown to mediate ERK activation. Whether impairment of PKC signaling may in fact explain the inhibition of neurotrophin response in BDV-infected neurons is currently under study.

PKC has long been suggested to play important roles in modulating synaptic efficacy, in particular through the regulation of vesicular cycling and neurotransmitter release [49]. Mice knockout for PKC-γ exhibit defects in LTP and learning [50], and impaired PKC activity has been associated with several neurobehavioral disorders and with neurodegenerative diseases such as Alzheimer's disease [51,52]. Thus, changes in PKC signaling and in presynaptic potentiation observed in BDV-infected neurons could critically affect learning and behavior. Our findings provide a molecular basis for the neurobehavioral and cognitive deficits that are associated with BDV persistence in the CNS.

## Materials and Methods

### Primary culture and virus.

Hippocampal neurons were prepared from newborn rats and infected with cell-free BDV (strain He/80), as described in [22]. BDV infection of neurons was verified by immunofluorescence for each experiment [17]. Neuronal cultures contained more than 80% neurons, as assessed by staining with neuron-specific markers, and all neurons were infected at the time experiments were performed (unpublished data).

### Antibodies and reagents.

We used: mouse monoclonal antibodies to the luminal domain of synaptotagmin I (Syt-ecto, clone 604.1, Synaptic Systems, Goettingen, Germany), Munc18 (Transduction Laboratories, Lexington, Kentucky, United States), NMDA receptor type 1 (NR1, clone 54.1; PharMingen, San Diego, California, United States), phospho-serine (Qiagen SA, Courtaboeuf, France), PKCα (clone MC5, sc-80; Santa Cruz Biotechnology, Santa Cruz, California, United States); rabbit polyclonals to the cytoplasmic domain of synaptotagmin I (Total-Syt; Synaptic Systems), phospho-MARCKS (Ser152/156, a site specifically phosphorylated by PKC; Cell Signaling Technology, Danvers, Massachusetts, United States), MARCKS (Chemicon), PKCɛ (Upstate, Charlottesville, Virginia, United States), PKCγ and PKCδ (sc-211 and sc-937, Santa Cruz Biotechnology). Phospho-Munc18 antibody was a gift from A. Morgan (University of Liverpool, Liverpool, United Kingdom). Antibodies specific for synapsin I phosphorylated by CaMK II at site 3 (clone RU19), or by PKA and CaMK I at site 1 (clone G257) were provided by P. Greengard (The Rockefeller University, New York, New York). All other antibodies have been described in [17]. Pharmacological agents were used at the following concentrations: 500 nM PMA, 50 μM forskolin and 1 μM tetrodotoxin (TTX; Sigma-Aldrich, Lyon, France), 200 nM Ro-31–8220 (Bis), a PKC inhibitor, and 20 μM H89, a PKA inhibitor (Calbiochem, San Diego, California, United States), 50 μM of NMDA receptor blocker D-(-)-2-amino-5-phosphopentanoic acid (APV) and 20 μM of AMPA/kainate receptor blocker 6-cyano-7-nitroquinoxaline-2,3-dione disodium (CNQX; Tocris Bioscience, Bristol, United Kingdom ). Botulinum B toxin (30 mM) was a gift from M. Popoff (Institut Pasteur, Paris, France).

### Exo-endocytotic assay.

The procedure for the analysis of SV recycling was adapted from a previously described method [20,53]. Experiments were performed at 37 °C in Tyrode's solution (119 mM NaCl/5 mM KCl/2 mM CaCl_2_/2 mM MgCl_2_/25 mM Hepes/30 mM glucose). Recycling SV were labeled with Syt-ecto, incubated on living neurons. Spontaneous SV recycling was measured in Tyrode's solution containing TTX, APV, and CNQX. Evoked SV recycling was measured in high-K^+^ Tyrode's solution (equimolar substitution of 70 mM KCl for NaCl) containing APV and CNQX, to prevent recurrent activity due to endogenous glutamate release. Other measurements were done after stimulation with pulses of high-K^+^ Tyrode's solution, or with modified Tyrode's solutions containing: glycine (200 μM glycine, 20 μM bicuculline, 1 μM strychnine), PMA, or forskolin. At the end of stimulation, neurons were allowed to rest for 10 min in the incubator, prior to measurement of post-stimulus spontaneous SV recycling. Neurons were then processed for immunocytochemistry, as described in [17]. Binding of Syt-ecto and total-Syt was revealed with anti mouse-Cy3 and anti rabbit-FITC secondary antibodies, respectively.

### Image acquisition and analysis.

Images were acquired using an epifluorescence microscope (Zeiss Axiovert 200M; Le Pecq, France ) equipped with a Roper Scientific Coolsnap HQ camera and a 63× oil immersion objective (Roper Scientific, Trenton, New Jersey, United States). Acquisition and analysis were carried out using SimplePCI (Compix, Hamamatsu Photonics Management Corporation, Delaware, United States). Coverslips were coded so that the investigator was blind to experimental conditions. Puncta with a fluorescence intensity in the total-Syt channel exceeding a threshold set above background were identified as synapses based on their localization at neuronal contacts. For each synapse, we measured mean fluorescence intensities for Cy3 (F1) and FITC (F2). After background subtraction, the ratio of fluorescence intensities F1/F2 was calculated, corresponding to normalized presynaptic activity.

### Calculation of presynaptic activities.

In order to compare a population of synapses, we plotted F1/F2 ratios measured at individual synapses against their frequency of occurrence to obtain frequency distribution plots (not shown). We then calculated the integral of the frequency distribution to obtain the cumulative probability distribution curves, shown in Figures 2, 3, and 5. A shift to the right indicates an increase in the number of synapses with high SV recycling. For the analysis of SV recycling from several independent experiments, we calculated for each experiment the mean presynaptic activity. To analyze the impact of treatments (Figures 3 and 5), means were expressed as the percentage of increase over means under basal conditions. Due to intrinsic variability between neuronal preparations, analysis was done by comparing pairwise mean presynaptic activities from control and BDV-infected cultures prepared and analyzed on the same day.

### Plasmids and transfection.

BDV P and BDV N were expressed in pCA vectors [54]. Transfections were performed with the rat Neuron Nucleofector Kit (Amaxa GmbH, Cologne, Germany), and transfected neurons were analyzed after 7 d. Transfection rates (verified by immunofluorescence) were always around 80%.

### PKC assays.

Basal and cofactor-stimulated PKC activities were measured using SignaTECT PKC Assay System (Promega, Charbonnières, France). Values for reactions lacking the substrate peptide were subtracted as blanks and specificity of reaction was verified by adding Bis.

### Subcellular fractionation.

Neurons were fractionated according to [37]. In brief, cells were lysed in 150-μl buffer A (12.5 mM Tris-HCl/2.5 mM EGTA/1 mM EDTA/100 mM NaF/5 mM DTT/300 μM PMSF/0.05% digitonin) and centrifuged. The soluble fraction was retained. The particulate fraction was washed and solubilized in 150 μl of buffer A containing 1% Triton X-100. Equal volumes of both fractions were subjected to Western blot.

### Cell extracts, Western blots, and immunoprecipitation.

Cell extracts and Western blots were performed as described in [17]. Densitometric analysis was performed using SCION Image (Scion, Frederick, Maryland, United States). Quantification of protein phosphorylation was carried out by measuring the density of the band corresponding to the phosphorylated protein normalized by the expression of the unphosphorylated protein, except for MARCKS phosphorylation which was normalized by β-tubulin III expression, due to inefficient stripping of phospho-MARCKS antibody binding. Results are expressed as percentage of increase over the mean of unstimulated controls. Immunoprecipitation of BDV P was performed according to [38].

### Data analysis.

Data are presented as mean ± standard error of the mean. The Kolmogorov-Smirnov test (KS) was used to compare the cumulative probability distributions of F1/F2 ratios. For experiments analyzing changes in mean presynaptic activities, statistical significance was assessed using the paired, two-tailed Student's *t* test. For the other data, statistical significance was assessed using the unpaired, two-tailed Student's *t* test.

## Supporting Information

### Accession Numbers

The SwissProt (http://www.expasy.org/sprot) accession numbers for proteins mentioned in the text are BDNF (P23363), BDV nucleoprotein (Q01552), BDV phosphoprotein (P26668), CaMK (P11275), EBV Zebra (P03206), ERK 1 and 2 (P21708 and P63086), HIV Nef (Q9QPN3), MARCKS (P30009), Munc13 (Q62768), Munc18/nSec1 (P61765), PKCα (P05696), PKCɛ (P09216), PKCγ (P63319), PKCδ (P09215), RACK1 (P63245), synapsin 1 (P09951), synaptotagmin 1 (P21707), TBK-1 (Q9WUN2), VAMP-2 (P63045). The GenBank (http://www.ncbi.nlm.nih.gov/Genbank) for the strain discussed in this paper is BDV strain He/80/FR (AJ311522).

## Abbreviations

BDV: Borna disease virus
CaMK: Ca2+/calmodulin-dependent kinase
CNS: central nervous system
ERK: extracellular-regulated kinase
GFP: green fluorescent protein
KS: Kolmogorov-Smirnov
LTP: long-term potentiation
MARCKS: myristoylated alanine-rich C kinase substrate
Munc18: Munc18–1/nSec1
N: nucleoprotein
NI: non-infected
NMDA: N-methyl-D-aspartate
P: phosphoprotein
PKA: protein kinase A
PKC: protein kinase C
PMA: phorbol 12-myristate 13-acetate
SV: synaptic vesicle
Syt-ecto: synaptotagmin I luminal domain–targeted monoclonal antibody
total-Syt: synaptotagmin I cytoplasmic domain–targeted polyclonal antibody
